# Presenteeism in front-line physicians involved in COVID-19-related clinical practice: a national survey of employed physician members of the Japan Medical Association

**DOI:** 10.1265/ehpm.22-00194

**Published:** 2023-02-03

**Authors:** Tomohiro Ishimaru, Toru Yoshikawa, Makoto Okawara, Michiko Kido, Yoshifumi Nakashima, Anna Nakayasu, Kokuto Kimori, Satoshi Imamura, Kichiro Matsumoto

**Affiliations:** 1 Department of Environmental Epidemiology, Institute of Industrial Ecological Sciences, University of Occupational and Environmental Health, Japan, Kitakyushu, Japan; 2 Research Center for Overwork-Related Disorders, National Institute of Occupational Safety and Health, Japan, Kawasaki, Japan; 3 Department of Obstetrics & Gynecology, Japanese Red Cross Medical Center, Tokyo, Japan; 4 Department of Psychiatry, Mitsui Memorial Hospital, Tokyo, Japan; 5 Exective Boards, Japan Medical Association, Tokyo, Japan; 6 Imamura Clinic, Tokyo, Japan; 7 President, Japan Medical Association, Tokyo, Japan

**Keywords:** COVID-19, Healthcare workers, Occupational health, Physicians, Presenteeism

## Abstract

**Background:**

The coronavirus disease 2019 (COVID-19) pandemic may have increased the rate of presenteeism among front-line physicians. Presenteeism is the term used to describe attendance at work despite ill health that would normally prompt rest or absence from work. This study aimed to examine the associations between COVID-19 clinical practice and presenteeism among physicians.

**Methods:**

A cross-sectional study was conducted from December 2021 to January 2022. The questionnaires were distributed to 21,737 employed physicians who were members of the Japan Medical Association. Presenteeism was measured by the Work Functioning Impairment Scale. Multiple logistic regression analysis was used to evaluate the association between COVID-19 clinical practice and presenteeism.

**Results:**

Overall, 3,968 participants were included in the analysis, and presenteeism was observed in 13.9% of them. The rate of presenteeism significantly increased with both the number of COVID-19 patients treated and the percentage of work time spent treating these patients (both *P* values for trend < 0.001). In comparison to those not currently engaged in the treatment of COVID-19 patients, presenteeism was significantly higher among front-line (adjusted odds ratio [aOR] = 1.71, 95% confidence interval [CI]: 1.16–2.53) and second-line physicians supporting those in the front-line (aOR = 1.45, 95% CI: 1.17–1.78). There was no association between involvement in COVID-19 vaccination services and presenteeism.

**Conclusions:**

The burden on front-line and second-line physicians in COVID-19 clinical practice must be minimized. Employed physicians also need to recognize the importance of communicating with their workplaces about presenteeism.

**Supplementary information:**

The online version contains supplementary material available at https://doi.org/10.1265/ehpm.22-00194.

## Introduction

Presenteeism is defined as attendance at work despite ill health that would normally prompt rest or absenteeism [1]. Healthcare professionals frequently experience presenteeism [2]. The major reasons for presenteeism are the desire not to burden colleagues and the lack of adequate cover [3]. A US study reported that half of clinical residents had worked with flu-like symptoms at least once in the previous year [4]. Physicians have to deal with excessive workloads, frequent overnight shifts and callouts, and voluminous paperwork [5]. Among physicians, presenteeism is associated with concerns about sick leave [6], and affects the quality of care and rate of medical errors [7].

The COVID-19 pandemic may have increased presenteeism among physicians. Studies of presenteeism during the COVID-19 pandemic have mainly focused on general workers [8–10] and healthcare staff [11, 12], and few have focused on physicians. A previous study of general workers showed that the COVID-19 pandemic was associated with psychological distress because of the requirement to perform unfamiliar tasks and work during lockdown, as well as concerns about employment instability [9]. Physicians also have to manage shortages of personal protective equipment, isolated and infected colleagues, and insufficient evidence about treatment for COVID-19. All these led to unfamiliar circumstances during the COVID-19 pandemic, especially for front-line physicians [13, 14]. We hypothesized that front-line physicians would experience higher rates of presenteeism as these conditions worsened. The purpose of this study was to examine the associations between COVID-19 and presenteeism among physicians.

## Methods

### Study design and participants

This cross-sectional study was based on the third national employed physician survey (2021); the survey is conducted every 6 years by the Japan Medical Association (JMA). The JMA was founded in Japan in 1916 as a private academic organization to support the medical activities of physicians. It is a professional organization that physicians join as individuals. Details of the first survey (2009) have been published elsewhere [15]. For the current survey, questionnaires were distributed and collected between December 9, 2021 and January 31, 2022. During the study period, which dovetailed with the “sixth wave” of COVID-19, the number of COVID-19 patients and deaths in Japan reached a peak [16]. Only employed physician members of the JMA were eligible for the study (a total of 72,859 physicians). The questionnaires were distributed by mail to 21,737 physicians; first, 10,000 physicians were randomly sampled, and then 11,737 physicians in their 20s and 30s were sampled in full. The latter sample was conducted because of the small number of respondents in their 20s and 30s in a previous survey [15]. Participants either returned the completed questionnaire by mail or completed it online via a QR code. A reminder was sent once during the survey period.

### Presenteeism (dependent variable)

Presenteeism was measured by the Work Functioning Impairment Scale (WFun) [17]. WFun was originally developed in Japan, and its validity and reliability have previously been assured [17]. The results show high correlations with other instruments for measuring presenteeism [1, 17, 18]. The WFun consists of seven items about work functioning impairment caused by ill health in the past 30 days. For each question, respondents choose from five numerical answer options, i.e., 1 (*low*) to 5 (*high*). The total WFun score therefore ranges from 7 to 35 points. A WFun score of ≥21 is associated with an increased risk of presenteeism [19], and a score of ≥25 is associated with an increased risk of sick leave [6]. Following previous studies, the cutoff for presenteeism used in this study was 21 points [7, 20].

### COVID-19-related clinical practice (independent variables)

COVID-19-related clinical practice was evaluated using four questions, based on previous studies [21–23]. The first question concerned the approximate number of COVID-19 patients treated by the respondent (category options: none; 1–10; 11–50, ≥51 patients). The second question concerned the percentage of time spent treating COVID-19 patients during work hours (category options: none; 1–9; 10–24, ≥25%). The third question concerned current direct contact with COVID-19 patients. Respondents in direct contact with COVID-19 patients were classified as front-line physicians (“major engagement”), while those in indirect contact with COVID-19 patients were classified as support staff for front-line workers or second-line physicians (“minor engagement”). All other respondents were classified as back-line physicians (“no engagement”). The last question was whether respondents were involved in COVID-19 vaccination services, with possible responses of yes or no.

### Covariates

Information on sex, age, specialty, hospital beds, work settings, hospital region and working conditions were extracted from the questionnaire. These covariates were selected based on a similar physician survey in the United States [24]. Specialty was categorized as follows: internal medicine, surgery, pediatrics, obstetrics and gynecology, psychiatry, or other. Work setting was categorized as academic/university or other. Hospital beds were classified into four categories: <100, 100–199, 200–499, and ≥500 beds. Hospital region used zip code. Regions with few respondents were combined with neighboring areas to ensure at least 10% in each category, and eventually classified into six regions. There were four variables included in working conditions in the last month: (1) experience of overwork, (2) amount of off-duty, (3) amount of overnight work, and (4) amount of on-call.

### Statistical analysis

We excluded questionnaires with missing outcomes or independent variable data from the analysis. Working conditions variables were dichotomized: (1) overwork (40 hours or more than the contracted time), (2) off-duty on 4 days or fewer, (3) overnight work on 4 days or more, and (4) on-call 5 days or more, all in the last month. The amount and percentage of COVID-19 clinical practice was calculated descriptively by each variable for each set of working conditions. We used chi-square tests for trends to estimate the *P* values for trend for each working conditions variable with COVID-19 clinical practice. Logistic regression analysis was used to evaluate the association between COVID-19 clinical practice and presenteeism. There were three models: Model 1 was univariate; Model 2 was adjusted for sex, age, specialty, hospital beds, work setting, and hospital region; and Model 3 was also adjusted for working conditions variables (overwork, off-duty, overnight, and on-call). We used trend analysis to evaluate the linear relationships based on logistic regression analysis with ordinal numbers assigned to each category. All *P* values were two-sided, and statistical significance was set at *P* < 0.05. We used Stata/SE software (ver. 16.1; StataCorp, College Station, TX, USA) for the analysis.

### Ethics

Participation was voluntary and the data were anonymized; therefore, the requirement for written informed consent was waived. There was no financial incentive for participation in the study, which was approved by the Research Ethics Committee of the National Institute of Occupational Safety and Health of Japan (2021N32).

## Results

A total of 4,032 people responded to the survey (response rate: 18.5%), of whom 3,968 were included in the analysis. Table 1 shows the characteristics of the participants. The majority of the participants were men (72.6%). In total, 22.2% of the respondents were aged 24–29 years, while 27.2% were aged ≥60 years. Approximately one-third of the respondents specialized in internal medicine (30.0%) and worked in hospitals with more than 500 beds (33.9%); 20.5% worked at academic/university hospitals and 20.8% at hospitals in the Kanto region. Around one-third of respondents had been overworked (had worked 40 hours or more than over their contracted time) (31.6%), off-duty for 4 days or fewer (31.1%) and worked overnight for 4 days or more (31.2%) in the last month. In total, 553 participants were classified into the high presenteeism group (13.9%).

**Table 1:** Characteristics of the study participants

**Variable (missing data)**	**N = 3,968 (%)**
Sex (10)	
Male	2,874 (72.6)
Female	1,084 (27.4)
Age, y (6)	
24–29	880 (22.2)
30–39	832 (21.0)
40–49	427 (10.8)
50–59	746 (18.8)
≥60	1,077 (27.2)
Specialty (3)	
Internal medicine	1,189 (30.0)
Surgery	663 (16.7)
Pediatrics, obstetrics and gynecology	418 (10.5)
Psychiatry	226 (5.7)
Other	1,469 (37.1)
Hospital beds (14)	
<100	423 (10.8)
100–199	728 (18.4)
200–499	1,461 (36.9)
≥500	1,342 (33.9)
Work setting (11)	
Academic/university	813 (20.5)
Other	3,144 (79.5)
Region (30)	
Hokkaido and Tohoku	462 (11.7)
Kanto	821 (20.8)
Chubu	672 (17.1)
Kansai	720 (18.3)
Chugoku and Shikoku	554 (14.1)
Kyushu and Okinawa	709 (18.0)
Overwork (worked ≥ 40 hours/month over their contracted time) (11)	1,255 (31.6)
Off-duty ≤ 4 days/month (41)	1,233 (31.1)
Overnight work ≥ 4 days/month (9)	1,237 (31.2)
On-call ≥ 5 days/month (17)	1,068 (26.9)
Presenteeism*	553 (13.9)

*Defined as a score of ≥21 points on the Work Functioning Impairment Scale.

Table 2 shows the amount and percentage of COVID-19-related clinical practice by working conditions. Fewer than 10% of the physicians were actively engaged in COVID-19 clinical practice; 342 (8.6%) treated ≥51 COVID-19 patients, 103 (2.6%) spent at least 25% of their work time with these patients, and 198 (5.0%) were currently involved in the front-line treatment of COVID-19 patients. These COVID-19-related clinical practices showed a trend to increase overwork, overnight work, and on-call and decrease off-duty time. Approximately two-thirds of the respondents (n = 2,715, 68.5%) were involved in providing COVID-19 vaccination services. Vaccination services were significantly associated with high overnight work, but not with other working conditions.

**Table 2:** Amount and percentage of COVID-19-related clinical practice by working conditions

**Variable (missing data)**	**Total**	**Overwork** **≥40 hours/month**	**Off-duty** **≤4 days/month**	**Overnight work** **≥4 days/month**	**On-call** **≥5 days/month**
	**N = 3,968**	**n = 1,255 (31.6%)**	**n = 1,233 (31.1%)**	**n = 1,237 (31.2%)**	**n = 1,068 (26.9%)**
Approximately how many COVID-19 patients have you ever treated? (6)					
None	1,085	230 (21.2)	253 (23.6)	197 (18.2)	217 (20.1)
1–10	1,781	587 (33.0)	590 (33.4)	586 (33.0)	545 (30.7)
11–50	754	284 (37.7)	252 (33.6)	306 (40.6)	199 (26.5)
≥51	342	154 (45.0)	138 (40.5)	147 (43.0)	107 (31.5)
*P* value for trend*		<0.001	<0.001	<0.001	<0.001
What percentage of your working hours are spent treating COVID-19 patients? (16)					
None	1,531	400 (26.2)	417 (27.6)	375 (24.5)	359 (23.5)
1%–9%	1,939	665 (34.4)	648 (33.7)	667 (34.5)	574 (29.7)
10%–24%	379	136 (35.9)	123 (32.5)	150 (39.6)	100 (26.4)
≥25%	103	50 (48.5)	42 (40.8)	43 (41.7)	32 (31.1)
*P* value for trend*		<0.001	<0.001	<0.001	0.001
What is your current level of engagement with COVID-19 patients? (11)					
No engagement	2,349	594 (25.3)	630 (27.1)	608 (25.9)	557 (23.8)
Minor engagement (second-line)	1,410	571 (40.6)	513 (36.6)	533 (37.8)	448 (31.9)
Major engagement (front-line)	198	90 (45.5)	88 (44.9)	95 (48.0)	63 (31.8)
*P* value for trend*		<0.001	<0.001	<0.001	<0.001
Have you ever been involved in COVID-19 vaccination services? (7)					
No	1,246	382 (30.7)	374 (30.5)	332 (26.6)	323 (26.0)
Yes	2,715	872 (32.2)	859 (31.9)	905 (33.4)	745 (27.5)
*P* value*		0.373	0.383	<0.001	0.344

*Chi-square tests for trend

Table 3 summarizes the associations between COVID-19 clinical practice and presenteeism. The results of the statistical tests showed similar trends in all models, although the odds ratios (ORs) were relatively attenuated in Model 3. Participants who had treated more COVID-19 patients were more likely to be classified into the high presenteeism group (*P* value for trend < 0.001). Similarly, presenteeism was higher among participants who spent a higher percentage of their working hours treating COVID-19 patients (*P* value for trend < 0.001). Compared with those not currently engaged in the treatment of COVID-19 patients, presenteeism was significantly higher among front-line (adjusted odds ratio [aOR] = 1.71, 95% confidence interval [CI]: 1.16–2.53) and second-line (aOR = 1.45, 95% CI: 1.17–1.78) physicians in Model 3. There was no association between involvement in COVID-19 vaccination services and presenteeism. The associations between covariates and presenteeism are provided in Supplemental Table 1.

**Table 3:** Associations between COVID-19-related clinical practice and presenteeism

**Variable (missing data)**	Presenteeism*	**Model 1**			**Model 2**			**Model 3**		
			
	**n (%)**	**OR**	**(95% CI)**	***P* value**	**OR**	**(95% CI)**	***P* value**	**OR**	**(95% CI)**	***P* value**
Approximately how many COVID-19 patients have you ever treated? (6)										
None	85 (7.8)	1.00	-	-	1.00	-	-	1.00	-	-
1–10 patients	259 (14.5)	2.00	(1.55–2.59)	<0.001	1.76	(1.34–2.30)	<0.001	1.61	(1.23–2.12)	0.001
11–50 patients	133 (17.6)	2.52	(1.89–3.37)	<0.001	2.05	(1.51–2.80)	<0.001	1.88	(1.37–2.58)	<0.001
≥51 patients	76 (22.2)	3.36	(2.40–4.71)	<0.001	2.84	(1.98–4.07)	<0.001	2.44	(1.68–3.52)	<0.001
*P* value for trend^†^				<0.001			<0.001			<0.001
What percentage of your working hours are spent treating COVID-19 patients? (16)										
None	156 (10.2)	1.00	-	-	1.00	-	-	1.00	-	-
1%–9%	306 (15.8)	1.65	(1.35–2.03)	<0.001	1.56	(1.26–1.93)	<0.001	1.43	(1.15–1.78)	0.001
10%–24%	68 (17.9)	1.93	(1.41–2.63)	<0.001	1.86	(1.34–2.56)	<0.001	1.70	(1.22–2.36)	0.002
≥25%	23 (22.3)	2.53	(1.55–4.15)	<0.001	2.37	(1.43–3.93)	<0.001	1.94	(1.15–3.25)	0.013
*P* value for trend^†^				<0.001			<0.001			<0.001
What is your current level of engagement with COVID-19 patients? (11)										
No engagement	261 (11.1)	1.00	-	-	1.00	-	-	1.00	-	-
Minor engagement (second-line)	247 (17.5)	1.70	(1.41–2.05)	<0.001	1.57	(1.29–1.93)	0.001	1.45	(1.17–1.78)	0.001
Major engagement (front-line)	45 (22.7)	2.35	(1.65–3.36)	<0.001	1.94	(1.33–2.84)	<0.001	1.71	(1.16–2.53)	0.007
*P* value for trend^†^				<0.001			<0.001			<0.001
Have you ever been engaged in COVID-19 vaccination services? (7)										
No	176 (14.1)	1.00	-	-	1.00	-	-	1.00	-	-
Yes	377 (13.9)	0.98	(0.81–1.19)	0.840	0.96	(0.79–1.18)	0.697	0.94	(0.77–1.16)	0.560

OR: odds ratio; CI: confidence interval.

*Defined as a score of ≥21 points on the Work Functioning Impairment Scale.

^†^Based on logistic regression analysis with ordinal numbers assigned to each category.

Model 1: Univariate analysis.

Model 2: Adjusted for sex, age, specialty, hospital beds, work setting, and region.

Model 3: Additionally adjusted for overwork, off-duty, overnight work, and on-call.

## Discussion

To the best of our knowledge, this is the first study to assess presenteeism among front-line physicians involved in COVID-19-related clinical practice. Presenteeism was observed in 13.9% of the respondents, which is the same as in the previous national employed physician survey in 2016 [25]. This indicates that the COVID-19 pandemic itself may not have exacerbated overall physician presenteeism. A dose-response relationship was seen, and the presenteeism rate increased with the number of COVID-19 patients treated and the percentage of work time spent treating these patients. The presenteeism rate was higher among both front-line physicians engaged in COVID-19 clinical practice and second-line physicians who supported them than other physicians. In this study, around 5% of physicians were classified as first-line for COVID-19 clinical practice, but if second-line physicians were included, the group was around 40% of the total. These findings suggest that a significant number of physicians were engaged in COVID-19-related clinical practice despite ill health that would normally prompt rest or absenteeism.

In this study, the presenteeism rate was higher among physicians frequently engaged in COVID-19-related clinical practice. Previous studies reported that front-line physicians frequently experience burnout and depression [24, 26]. Patient contact, high workload, mandatory overtime, fear of unemployment, and especially mental burden are predictors of presenteeism [27]. Our results suggest that presenteeism may be more responsive to the number of COVID-19 patients treated than the time spent treating these patients. There are several possible explanations for this result. First, the large number of patients means that the time spent on each was limited and decisions had to be made quickly. Second, being on-call at night and weekends would have increased the volume of patients seen; both of these factors negatively affect physician mental health [28, 29]. In this study, the adjusted model that included working conditions (Model 3) showed relatively lower ORs compared with the model that did not include them (Model 2), suggesting that working conditions are one of potential factors influencing presenteeism.

Presenteeism can also be an issue for second-line physicians engaged in COVID-19-related clinical practice. Our results showed that second-line physicians had high presenteeism rates, although not as high as front-line physicians (aOR: 1.45 vs. 1.71). Previous studies examining the mental health of front- and second-line physicians engaged in COVID-19-related clinical practice have reported contradictory findings: some reported that front line physicians had poorer mental health [30], while others reported no difference [31, 32]. These findings could be applied to mental health-related presenteeism [33]. It is plausible that the workload for second-line physicians may be increased by the necessity to treat both COVID-19 and non-COVID-19 patients. Thus, interventions are needed not only for front-line physicians but also to address presenteeism among the second-line physicians who support them.

The majority of physicians (68.5%) in our study were involved in COVID-19 vaccination services. Although the rollout of the COVID-19 vaccine was initially delayed in Japan [34], the COVID-19 mass vaccination program rapidly progressed because of the efforts of healthcare workers, and has slowed the spread of infection [35, 36]. We found that involvement in vaccination services was not associated with presenteeism. COVID-19 vaccinations were sometimes offered at outdoor triage stations and emergency medical tents outside the main hospital buildings in other countries [23], but this was rarely seen in Japan. In other words, the working conditions for physicians engaged in COVID-19 vaccination services in Japan are relatively favorable and do not contribute to increased presenteeism. This study also found no association between engagement in COVID-19 vaccination services and overwork (Table 2).

This study has various implications with respect to presenteeism among physicians engaged in COVID-19-related clinical practice. First, the burden on front- and second-line physicians associated with the large number of COVID-19 patients must be minimized. For example, controlling workload, providing adequate rest periods and sufficient personal protective equipment are crucial for physicians to effectively treat COVID-19, as is family support [37]. A large proportion of tasks completed by physicians can be performed by non-physicians; this situation contributes to presenteeism [38]. It is necessary to identify the types of COVID-19 clinical practice that can be handed over to other staff groups. Second, it is essential that workplaces make plans to cover for front-line physicians who become ill. Physicians also need to recognize the importance of communicating with their workplaces about ill health [39].

There were several limitations to this study. First, the response rate was relatively low (18.5%). Physicians in their 20s and 30s are generally busy and thus show lower response rates [15]. Our results may therefore have been affected by lack of participation from physicians who are particularly busy and have high levels of presenteeism. To reduce bias due to a skewed age distribution, we used a full rather than random sampling method for that population; we also sent a reminder during the survey. Another limitation was the cross-sectional design, which precluded the establishment of causality. However, engaging in COVID-19-related clinical practice because of high presenteeism seems unlikely.

## Conclusion

We identified a dose-response relationship between COVID-19-related clinical practice and presenteeism among physicians. The rate of presenteeism was highest among front-line physicians, but it was also relatively high among the second-line physicians who support them. The burden imposed on front- and second-line physicians by the large number of COVID-19 patients must therefore be minimized. Employed physicians also need to recognize the importance of communicating with their workplaces about presenteeism; presenteeism not only affects sick leave but also the quality of care and medical errors.

## Abbreviations

COVID-19: Coronavirus disease 2019
WFun: Work Functioning Impairment Scale

## Supplementary information

**Additional file 1:** **Supplemental Table 1.** Associations between covariates and presenteeism.
