# Supplementary material for: Presenteeism in front-line physicians involved in COVID-19-related clinical practice: a national survey of employed physician members of the Japan Medical Association
Source: Environ Health Prev Med. 2023 Feb 3;28:13. doi: 10.1265/ehpm.22-00194 (PMC9922563; doi:10.1265/ehpm.22-00194)
Supplement: Supplementary file 1 — Additional file 1: Supplemental Table 1. Associations between covariates and presenteeism. [file ehpm-28-013-s001.docx]

Supplemental Table 1. Associations between covariates and presenteeism

| **Variable (missing data)** |  | **Presentee-ism*** |  | **Model 1** | | |  | **Model 2** | | |  | **Model 3** | | |
| --- | --- | --- | --- | --- | --- | --- | --- | --- | --- | --- | --- | --- | --- | --- |
|  |  | **n (%)** |  | **OR** | **(95% CI)** | ***P* value** |  | **OR** | **(95% CI)** | ***P* value** |  | **OR** | **(95% CI)** | ***P* value** |
| Sex |  |  |  |  |  |  |  |  |  |  |  |  |  |  |
| Male |  | 409 (14.2) |  | 1.00 | - | - |  | 1.00 | - | - |  | 1.00 | - | - |
| Female |  | 142 (13.1) |  | 0.91 | (0.74–1.12) | 0.359 |  | 0.70 | (0.56–0.87) | 0.001 |  | 0.81 | (0.65–1.02) | 0.075 |
| Age, y |  |  |  |  |  |  |  |  |  |  |  |  |  |  |
| 24–29 |  | 155 (17.6) |  | 1.00 | - | - |  | 1.00 | - | - |  | 1.00 | - | - |
| 30–39 |  | 158 (19.0) |  | 1.10 | (0.86–1.40) | 0.461 |  | 1.10 | (0.83–1.46) | 0.524 |  | 1.16 | (0.87–1.56) | 0.309 |
| 40–49 |  | 60 (14.1) |  | 0.77 | (0.55–1.06) | 0.103 |  | 0.78 | (0.54–1.11) | 0.168 |  | 0.80 | (0.55–1.17) | 0.245 |
| 50–59 |  | 105 (14.1) |  | 0.77 | (0.59–1.00) | 0.052 |  | 0.76 | (0.56–1.05) | 0.094 |  | 0.79 | (0.57–1.11) | 0.173 |
| ≥ 60 |  | 74 (6.9) |  | 0.35 | (0.26–0.46) | < 0.001 |  | 0.32 | (0.23–1.05) | < 0.001 |  | 0.40 | (0.27–0.59) | < 0.001 |
| Specialty |  |  |  |  |  |  |  |  |  |  |  |  |  |  |
| Internal medicine |  | 158 (13.3) |  | 1.00 | - | - |  | 1.00 | - | - |  | 1.00 | - | - |
| Surgery |  | 84 (12.7) |  | 0.95 | (0.71–1.26) | 0.705 |  | 0.97 | (0.72–1.30) | 0.834 |  | 0.87 | (0.64–1.17) | 0.357 |
| Pediatrics, obstetrics and gynecology |  | 44 (10.5) |  | 0.77 | (0.54–1.09) | 0.143 |  | 0.74 | (0.51–1.07) | 0.104 |  | 0.69 | (0.47–1.01) | 0.056 |
| Psychiatry |  | 32 (14.2) |  | 1.08 | (0.72–1.62) | 0.725 |  | 1.04 | (0.68–1.59) | 0.850 |  | 1.37 | (0.89–2.11) | 0.155 |
| Other |  | 234 (15.9) |  | 1.24 | (0.99–1.54) | 0.056 |  | 1.02 | (0.79–1.31) | 0.904 |  | 1.15 | (0.89–1.49) | 0.291 |
| Hospital beds |  |  |  |  |  |  |  |  |  |  |  |  |  |  |
| < 100 |  | 42 (9.9) |  | 1.00 | - | - |  | 1.00 | - | - |  | 1.00 | - | - |
| 100–199 |  | 83 (11.4) |  | 1.17 | (0.79–1.73) | 0.439 |  | 1.12 | (0.75–1.68) | 0.572 |  | 1.10 | (0.73–1.65) | 0.648 |
| 200–499 |  | 209 (14.3) |  | 1.51 | (1.07–2.15) | 0.020 |  | 1.18 | (0.82–1.71) | 0.369 |  | 0.98 | (0.67–1.42) | 0.895 |
| ≥ 500 |  | 217 (16.2) |  | 1.75 | (1.23–2.48) | 0.002 |  | 0.98 | (0.65–1.48) | 0.938 |  | 0.72 | (0.48–1.10) | 0.134 |

OR: odds ratio; CI: confidence interval.

* Defined as a score of ≥ 21 points on the Work Functioning Impairment Scale.

Model 1: Univariate analysis.; Model 2: Adjusted for sex, age, specialty, hospital beds, work setting, and region.; Model 3: Additionally adjusted for overwork, off-duty, overnight work, and on-call.

Supplemental Table 1. Associations between covariates and presenteeism (Cont.)

| **Variable (missing data)** |  | **Presentee-ism*** |  | **Model 1** | | |  | **Model 2** | | |  | **Model 3** | | |
| --- | --- | --- | --- | --- | --- | --- | --- | --- | --- | --- | --- | --- | --- | --- |
|  |  | **n (%)** |  | **OR** | **(95% CI)** | ***P* value** |  | **OR** | **(95% CI)** | ***P* value** |  | **OR** | **(95% CI)** | ***P* value** |
| Work setting |  |  |  |  |  |  |  |  |  |  |  |  |  |  |
| Academic/university |  | 153 (18.8) |  | 1.00 | - | - |  | 1.00 | - | - |  | 1.00 | - | - |
| Other |  | 399 (12.7) |  | 0.63 | (0.51–0.77) | < 0.001 |  | 0.70 | (0.53–0.93) | 0.013 |  | 0.68 | (0.51–0.90) | 0.007 |
| Region |  |  |  |  |  |  |  |  |  |  |  |  |  |  |
| Hokkaido and Tohoku |  | 66 (14.3) |  | 1.00 | - | - |  | 1.00 | - | - |  | 1.00 | - | - |
| Kanto |  | 120 (14.6) |  | 1.03 | (0.74–1.42) | 0.872 |  | 0.97 | (0.70–1.37) | 0.879 |  | 1.02 | (0.72–1.44) | 0.935 |
| Chubu |  | 97 (14.4) |  | 1.01 | (0.72–1.42) | 0.944 |  | 1.02 | (0.72–1.44) | 0.907 |  | 1.07 | (0.75–1.51) | 0.724 |
| Kansai |  | 108 (15.0) |  | 1.06 | (0.76–1.48) | 0.735 |  | 1.04 | (0.74–1.46) | 0.812 |  | 1.11 | (0.78–1.57) | 0.563 |
| Chugoku and Shikoku |  | 63 (11.4) |  | 0.77 | (0.53–1.11) | 0.165 |  | 0.78 | (0.53–1.13) | 0.189 |  | 0.78 | (0.53–1.14) | 0.201 |
| Kyushu and Okinawa |  | 94 (13.3) |  | 0.92 | (0.65–1.29) | 0.617 |  | 0.91 | (0.65–1.29) | 0.600 |  | 0.90 | (0.63–1.28) | 0.569 |
| Overwork (Work over their contracted time) | | | | | | | | | | | | | | |
| < 40 hours/month |  | 275 (10.2) |  | 1.00 | - | - |  | 1.00 | - | - |  | 1.00 | - | - |
| ≥ 40 hours/month |  | 278 (22.2) |  | 2.51 | (2.09–3.01) | < 0.001 |  | 2.39 | (1.96–2.90) | < 0.001 |  | 2.13 | (1.74–2.62) | < 0.001 |
| Off-duty |  |  |  |  |  |  |  |  |  |  |  |  |  |  |
| > 4 days/month |  | 312 (11.6) |  | 1.00 | - | - |  | 1.00 | - | - |  | 1.00 | - | - |
| ≤ 4 days/month |  | 237 (19.2) |  | 1.82 | (1.51–2.18) | < 0.001 |  | 1.73 | (1.42–2.10) | < 0.001 |  | 1.40 | (1.14–1.72) | 0.001 |
| Overnight work |  |  |  |  |  |  |  |  |  |  |  |  |  |  |
| < 4 days/month |  | 344 (12.6) |  | 1.00 | - | - |  | 1.00 | - | - |  | 1.00 | - | - |
| ≥ 4 days/month |  | 209 (16.9) |  | 1.41 | (1.17–1.69) | < 0.001 |  | 1.08 | (0.88–1.33) | 0.475 |  | 0.98 | (0.79–1.21) | 0.841 |
| On-call |  |  |  |  |  |  |  |  |  |  |  |  |  |  |
| < 5 days/month |  | 358 (12.4) |  | 1.00 | - | - |  | 1.00 | - | - |  | 1.00 | - | - |
| ≥ 5 days/month |  | 193 (18.1) |  | 1.56 | (1.29–1.88) | < 0.001 |  | 1.58 | (1.28–1.96) | < 0.001 |  | 1.23 | (0.98–1.54) | 0.071 |

OR: odds ratio; CI: confidence interval.

* Defined as a score of ≥ 21 points on the Work Functioning Impairment Scale.

Model 1: Univariate analysis.; Model 2: Adjusted for sex, age, specialty, hospital beds, work setting, and region.; Model 3: Additionally adjusted for overwork, off-duty, overnight work, and on-call.
